# Focused Overview of *Mycobacterium tuberculosis* VapBC Toxin–Antitoxin Systems Regarding Their Structural and Functional Aspects: Including Insights on Biomimetic Peptides

**DOI:** 10.3390/biomimetics8050412

**Published:** 2023-09-06

**Authors:** Sung-Min Kang

**Affiliations:** College of Pharmacy, Duksung Women’s University, Seoul 01369, Republic of Korea; smkang@duksung.ac.kr

**Keywords:** *Mycobacterium tuberculosis*, toxin-antitoxin system, VapBC protein complex

## Abstract

Tuberculosis, caused by *Mycobacterium tuberculosis*, is a lethal infectious disease of significant public health concern. The rise of multidrug-resistant and drug-tolerant strains has necessitated novel approaches to combat the disease. Toxin–antitoxin (TA) systems, key players in bacterial adaptive responses, are prevalent in prokaryotic genomes and have been linked to tuberculosis. The genome of *M. tuberculosis* strains harbors an unusually high number of TA systems, prompting questions about their biological roles. The VapBC family, a representative type II TA system, is characterized by the VapC toxin, featuring a PilT N-terminal domain with nuclease activity. Its counterpart, VapB, functions as an antitoxin, inhibiting VapC’s activity. Additionally, we explore peptide mimics designed to replicate protein helical structures in this review. Investigating these synthetic peptides offers fresh insights into molecular interactions, potentially leading to therapeutic applications. These synthetic peptides show promise as versatile tools for modulating cellular processes and protein–protein interactions. We examine the rational design strategies employed to mimic helical motifs, their biophysical properties, and potential applications in drug development and bioengineering. This review aims to provide an in-depth understanding of TA systems by introducing known complex structures, with a focus on both structural aspects and functional and molecular details associated with each system.

## 1. Introduction

Tuberculosis, caused by the pathogenic bacterium *Mycobacterium tuberculosis*, ranks among the most lethal infectious diseases [1,2]. It holds a significant position on the list of global public health concerns, with an annual death toll exceeding 1.5 million [3,4]. The emergence of multidrug-resistant (MDR) and extensively drug-resistant (XDR) strains of *M. tuberculosis* presents a formidable challenge to current treatment approaches [5,6]. This situation highlights the urgent need for innovative methods to combat the evolving landscape of drug resistance. Furthermore, *M. tuberculosis* often exhibits drug tolerance, allowing it to survive even under antibiotic therapy [7,8]. Thus, the development of fresh strategies to address tuberculosis, rooted in emerging molecular mechanisms, is of utmost importance.

Toxin–antitoxin (TA) systems were initially identified as compact genetic modules typically located on plasmids. Their primary role was seen in safeguarding plasmids by triggering cell death in individuals lacking the plasmid-borne TA encoding genes [9]. TA loci are prevalent in prokaryotic genomes and have increasingly been associated with bacterial adaptive responses, notably impacting the progression of tuberculosis infections [10,11]. Subsequent investigations unveiled the presence of TA loci on bacterial and archaeal chromosomes, suggesting additional roles [12]. In times of stress, such as plasmid loss or bacteriophage infection, antitoxins can rapidly degrade. This degradation activates toxins, which selectively target crucial cellular processes, including DNA replication, cell wall synthesis, cell division, and translation. Consequently, these processes become vulnerable to toxin influence, leading to growth inhibition and eventual cell demise [13,14]. The growth inhibition inflicted by toxins can be countered by freshly synthesized antitoxins, suggesting that TA system activation may aid bacterial survival during adverse conditions, allowing them to persist until more favorable environmental conditions emerge [15,16].

The genome of *M. tuberculosis* strains is notable for harboring an unusually high number of TA systems, with the laboratory strain H37Rv alone encoding over 100 such modules, believed to play a role in its pathogenesis [17,18,19]. These systems consist of toxin–antitoxin pairs organized into operons and distributed widely throughout the bacterial genome [20,21]. Given the multitude of TA loci in the *M. tuberculosis* genome, several critical questions arise regarding their functional diversity [22]. These inquiries delve into understanding the mechanisms responsible for the proliferation of TA loci within the genome and the intricate regulation of their activity within such a complex system [23,24]. Moreover, they extend to questions regarding potential functional redundancy, the advantages these TA loci confer during infection, and the triggers that activate them [25,26]. It is noteworthy that *M. tuberculosis* exclusively features type II TA systems, with no other TA family identified within its genome [27,28]. This unique prevalence of type II TA systems invites further exploration into their potential specialized roles and evolutionary significance within the framework of *M. tuberculosis*.

There are seven known classes of TA systems, each with a different mode of action to inhibit the toxin [9,29]. Type I systems have a small anti-sense RNA antitoxin that forms a duplex with the toxin’s mRNA to inhibit toxin production [30]. Type II systems have a protein antitoxin that interacts with the toxin to form a complex in which the toxin is inactive. They often act as auto-repressors of their own transcription [31]. Type III antitoxins are RNAs that inactivate the toxin by forming a complex [27]. Type IV systems are represented by the antitoxin, which suppresses the toxicity of the toxin by stabilizing its targets [32,33,34]. Type V is represented by the GhoT-GhoS system, in which the antitoxin inhibits the toxin by specific cleavage of its mRNA [35]. Type VI systems use an antitoxin that serves as an adaptor protein to address the toxin to protease [36]. Type VII antitoxins neutralize the toxin through post-translational modification of the toxin, such as phosphorylation [37,38,39,40]. Among these TA systems, the virulence-associated proteins (VapBC) system, which is the focus of this paper, belongs to the type II category and stands out as one of the representative systems in this class [23,41]. The VapBC family is notable for the inclusion of the VapC toxin, which features a PilT N-terminal (PIN) domain and exhibits nuclease activity. Its cognate VapB protein acts as an antitoxin that inhibits the activity of the VapC toxin [42,43]. The VapC toxin cleaves RNA to suppress metabolic activity as part of a survival strategy, and this process requires the VapB antitoxin [21,44,45]. This also implies that their toxic activity must be tightly regulated in order not to be detrimental for bacterial survival [41,46].

The *M. tuberculosis* genome boasts an extraordinary proliferation of type II TA systems, with the VapBC system being particularly prominent [23]. Within the realm of type II TA systems, the toxins are characterized by their stability, often featuring antiparallel β-sheet cores [44]. On the other side, the antitoxins are also proteins but tend to possess more relaxed and flexible structures, rendering them susceptible to degradation [45]. Under normal circumstances, the antitoxin effectively curbs the activity of the toxin by forming a stable protein–protein complex. However, in the face of environmental stresses, this intricate equilibrium between toxin and antitoxin is disrupted [21]. The antitoxin succumbs to degradation under stress, thereby unleashing the free toxin to target its substrates, consequently leading to the establishment of a bacteriostatic state [46].

Notably, the active sites of VapC toxins house several conserved acidic residues, collectively forming a negatively charged cavity that facilitates coordination with divalent metal ions such as Mg^2+^ and Mn^2+^ [47,48]. On the other hand, the VapB antitoxins comprise two functional motifs: an N-terminal domain that binds to the promoter DNA of the TA operon, and a C-terminal domain that engages with the cognate VapC toxin, effectively nullifying its toxic impact [48]. These intricate interactions between toxins and antitoxins underscore the sophisticated regulatory mechanisms inherent to type II TA systems, providing insights into the finely tuned responses of bacteria to diverse environmental challenges.

In recent years, there has been a growing interest in utilizing peptides as a strategy to interfere with TA systems, particularly as potential targets for antibacterial interventions. TA systems are genetic modules present in many bacterial pathogens but notably absent in eukaryotic cells [21,45,46]. This fundamental difference suggests that compounds derived from TA systems could potentially offer reduced side effects in humans and increased specificity in targeting bacterial pathogens. These innovative approaches offer exciting prospects for the discovery of new antibacterial targets and the development of novel mechanisms distinct from the antibiotics currently used to treat tuberculosis and other bacterial infections. Researchers have been investigating the use of peptides that mimic the structure of the TA interface [49,50]. By designing peptides that can effectively bind to the binding interface of the TA complex, scientists aim to release the toxin, ultimately leading to bacterial death. This approach is particularly appealing because peptides can be customized to target specific bacterial pathogens, thanks to the unique structures of each TA system in bacteria.

**Figure 1:**
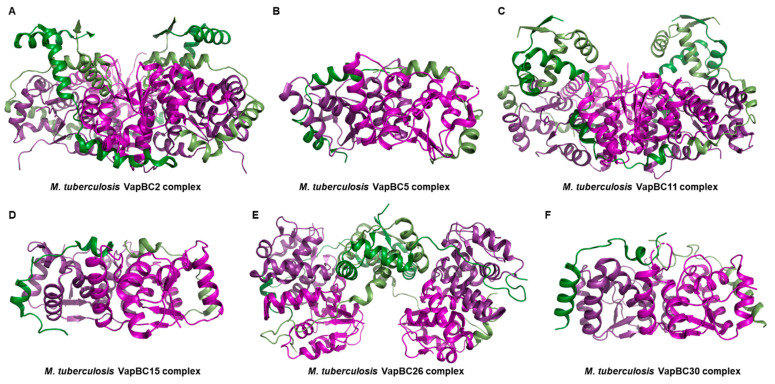
Complex structures of known *M. tuberculosis* VapBC systems. Structural figures were generated using PyMol (Version 2.5, Schrödinger, LLC, New York, NY, USA). The structures of each VapB antitoxin are depicted in varying tones of green, while the structures of each VapC toxin are depicted in varying tones of purple. (**A**) Overall view of *M. tuberculosis* VapBC2 complex (PDB ID 3H87). (**B**) Overall view of *M. tuberculosis* VapBC5 complex (PDB ID 3DBO). (**C**) Overall view of *M. tuberculosis* VapBC11 complex (PDB ID 6A7V). (**D**) Overall view of *M. tuberculosis* VapBC15 complex (PDB ID 4CHG). (**E**) Overall view of *M. tuberculosis* VapBC26 complex (PDB ID 5X3T). (**F**) Overall view of *M. tuberculosis* VapBC30 complex (PDB ID 4XGR). (**A**,**C**,**E**): Hetero-octameric assembly; (**B**,**D**,**F**): Hetero-tetrameric assembly.

As a structural biologist, the primary objective of this review is to offer an extensive comprehension of the TA systems by presenting an exposition of six known complex structures (Figure 1A–F). Specifically, these structures encompass *M. tuberculosis* VapBC2 (PDB ID 3H87) [51], *M. tuberculosis* VapBC5 (PDB ID 3DBO) [52], *M. tuberculosis* VapBC11 (PDB ID 6A7V) [53], *M. tuberculosis* VapBC15 (PDB ID 4CHG) [54], *M. tuberculosis* VapBC26 (PDB ID 5X3T) [49], and *M. tuberculosis* VapBC30 (PDB ID 4XGR) [50]. In addition to its structural focus, this review offers a comprehensive exploration of the functional and molecular intricacies associated with each TA system. This encompasses detailed investigations into the configurations and binding modes of toxins and antitoxins, their respective functional roles, and various potential applications. We aim for this comprehensive review of the six known complex structures presented in this article to serve as a valuable resource for researchers seeking a deep understanding of TA systems. Given that numerous review papers have already covered the general aspects of the VapBC system, our primary objective in this paper is to place greater emphasis on the structural dimension of the VapBC system. For instance, we refrain from revisiting discussions on common subjects such as the PIN domain and active site, which comprises several acidic, negatively charged residues, and is crucial for catalytic ribonuclease activity.

## 2. Focused Overviews on Structural and Functional Aspects

### 2.1. VapBC2 System

**Figure 2:**
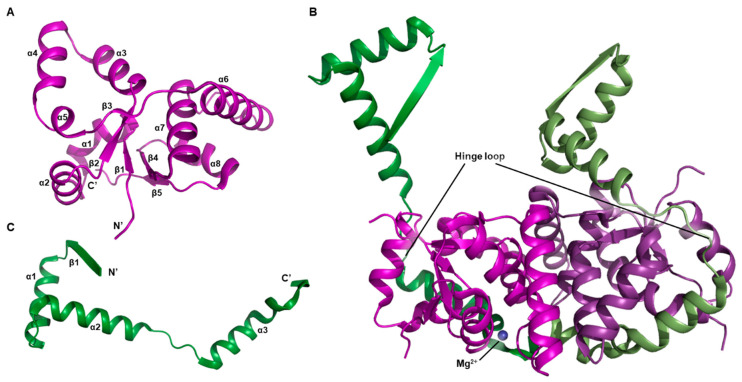
Unit structures belongs to *M. tuberculosis* VapBC2 system. Secondary structure nominations are displayed. (**A**) Overall architecture of VapC2 toxin. (**B**) Overall architecture of VapBC2 complex in hetero-tetrameric assembly. This asymmetric unit consists of two VapB2 antitoxin molecules and two VapC2 toxin molecules. Coordinate Mg^2+^ ion is also indicated. (**C**) Overall architecture of VapB2 antitoxin.

The overall structure of VapC2 toxin has a characteristic sandwich-like topology, which is typical of a canonical PilT N-terminus (PIN)-domain protein (Figure 2A) [55,56]. This structural framework unfolds through the coordination of five parallel β-strands intertwined with a symphony of eight α-helices. A notable observation arises from the complexities of the VapC2 dimer, where a subtle yet significant pseudo-twofold symmetry prevails, albeit marked by subtle conformational nuances, as artistically depicted in Figure 2B. However, a noteworthy aspect is that only one of the two VapC2 molecules forming the dimeric arrangement binds to Mg^2+^. As a result, this indicates that one of the toxin molecules assumes the role of a Mg^2+^-bound entity, while its dimeric partner remains unbound to Mg^2+^.

The structure of the VapB2 antitoxin is comprised of an N-terminal β-strand and three α-helices (Figure 2C). The N-terminal β1–α1–α2 region constitutes a ribbon–helix–helix (RHH) domain, which is a common structural motif in transcription factors that enables them to bind to DNA [57]. The long loop between α2 and α3 creates a flexible hinge, and a part of this hinge, along with the C-terminal a3 and a4, forms the interface with the toxin. In contrast to the toxin molecules, which have limited conformational differences, the antitoxins have significantly distinct conformations due to their varied hinges (Figure 2B). Moreover, the Mg^2+^-unbound toxin is wrapped more tightly by its proximal antitoxin than Mg^2+^-bound toxin is by the other antitoxin.

A hetero-octamer is formed by two hetero-tetrameric units related by a twofold symmetry, and the majority of interactions between the units occur within the VapB2–VapB2 dimeric interface (Figure 1A). The N-terminal β-strands of the two VapB2 molecules interact, completing the RHH motif by forming a dimer. An antiparallel sheet is formed by the β-strands from each VapB2. Additionally, the C-terminus of the VapB2 binds two toxins together [51].

### 2.2. VapBC5 System

**Figure 3:**
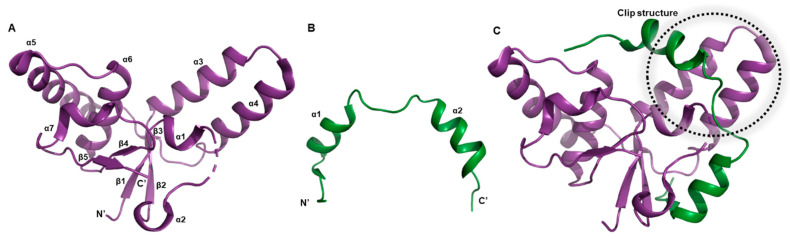
Unit structures belongs to *M. tuberculosis* VapBC5 system. Secondary structure nominations are displayed. (**A**) Overall architecture of VapC5 toxin. (**B**) Overall architecture of VapB5 antitoxin. A significant portion of the N-terminal region lacks electron density map, and only the region that closely binds to the toxin can be observed. (**C**) Overall architecture of VapBC5 complex in hetero-dimeric assembly. The dotted lines represent the clip structure, which consists of α3 and α4 helices.

The *M. tuberculosis* VapC5 toxin has a condensed α/β/α main domain (Figure 3A). This toxin also shows an additional feature-α clip structure constituted by two protruding α-helices (Figure 3C) [58]. The core domain, known for its compact nature, unfolds with elegance, encompassing a four-stranded parallel β-sheet (β2–β1–β3–β4) encircled by a delicate arrangement of five α-helices. The clip structure, consisting of α3 and α4, is connected to the core domain by two flexible stretches that appear as coils. These segments likely confer the necessary flexibility for binding to the antitoxin [59,60,61].

The structure of the VapB5 antitoxin reveals the absence of certain residues within the N-terminal segment, responsible for DNA binding (Figure 3B). VapB5 adopts a helical conformation, comprising two α-helices, namely α1 and α2, interconnected by an elongated and pliable loop. Notably, VapB5 engages with VapC5 at a substantially expansive and profound interface, originating between the core domain and the clip structure (Figure 3C).

The VapBC5 complex adopts a hetero-tetrameric arrangement facilitated by a twofold axis within the crystallographic symmetry, thereby establishing a relationship between two hetero-dimeric units (Figure 1B). Regarding its function, the VapC5 toxin has been demonstrated to exhibit Mg^2+^-dependent activity in nuclease assays, implying the necessity of Mg^2+^ ions for its enzymatic function. Empirical in vitro tests have substantiated that VapC5 possesses the capability to cleave general 150-nucleotide RNA molecules [52].

### 2.3. VapBC11 System

**Figure 4:**
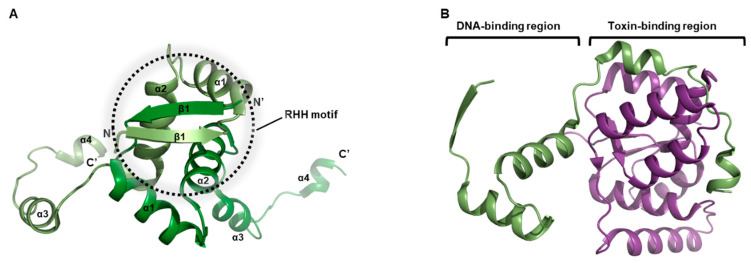
Unit structures belong to *M. tuberculosis* VapBC11 system. Secondary structure nominations are displayed. (**A**) Overall architecture of VapB11 antitoxin dimer. Location of RHH motif is denoted. (**B**) Overall architecture of VapB11 complex homodimer. N-terminal β1–α1–α2 domain of VapB11 is responsible for DNA-binding, and C-terminal α3 and α4 helices are responsible for VapC11 toxin binding.

The crystallographic depiction of the VapBC11 system reveals distinct high-resolution observations of both the N-terminal and C-terminal regions of the VapB11 antitoxin (Figure 4A). The N-terminal domain of VapB11 constitutes the formation of a RHH DNA-binding motif. By oligomerizing at the N-terminus, VapB11 establishes a dimeric configuration. Further interaction predominates in the remaining C-terminal segment, encompassing α3 and α4, which engages with the VapC11 toxin (as illustrated in Figure 4B). When the VapBC11 complex assembles, the activity of each toxin is counteracted by binding its corresponding antitoxin molecule. Notably, as VapC11 is recognized for its tRNA cleavage capability, investigation into the binding kinetics of tRNA with immobilized VapC11 has revealed a dissociation constant (*Kd*) of approximately 0.5 nM [53].

The interaction between VapB11 and VapC11 transpires with a 1:1 stoichiometry. Within the crystallographic symmetry of VapBC11, the intertwining of VapB11 and VapC11 leads to the formation of a hetero-octameric complex (Figure 1C). In solution, it is observed that two homodimers of VapB11, along with two homodimers of VapC11, interplay to craft a hetero-octameric assembly [53]. Moreover, surface plasmon resonance experiments have corroborated that VapB11 and VapC11 manifest interactions characterized by affinities within the nanomolar range [53].

### 2.4. VapBC15 System

The VapBC15 *M. tuberculosis* complex structure consists of an 80-residue VapB15 antitoxin and a 132-residue VapC15 toxin. These co-expressed proteins combine to form a hetero-tetrameric assembly. In the combined structure, each toxin monomer is bridged by an antitoxin monomer (Figure 1D).

**Figure 5:**
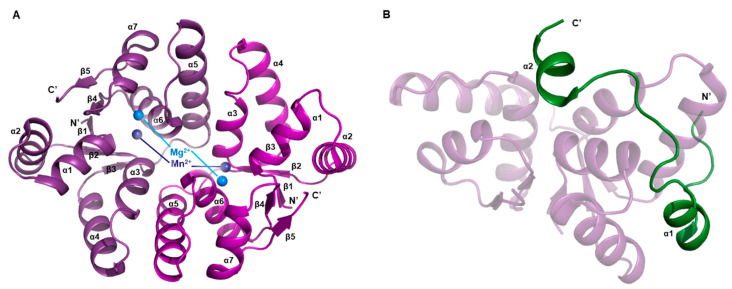
Unit structures belongs to *M. tuberculosis* VapBC15 system. Secondary structure nominations are displayed. (**A**) Overall architecture of VapC15 toxin dimer. Locations of Mg^2+^ and Mn^2+^ are represented by blue and indigo spheres respectively. (**B**) Overall architecture of VapB15 in VapBC15 complex homodimer. To provide a reference for the binding mode with VapC15, VapB15 and VapC15 are presented alongside, and VapC15 is treated transparently.

Within the dimeric arrangement of VapC15, an intriguing observation surfaces as two pairs of metal ions are detected (Figure 5A). The VapC15 toxin monomer presents a compact, globular structure encompassing an α/β/α fold, characteristic of PIN-domain proteins [55,56]. Structural analysis of an architecture reveals a composition of 12 secondary structure elements, specifically β1–α1–α2–β2–α3–α4–β3–α5–α6–β4–α7–β5. The pivotal central domain of VapC15 encompasses a five-stranded parallel β-sheet, sequenced as β3–β2–β1–β4–β5, flanked on one side by four α-helices (α1–α4) and on the other side by three α-helices (α5–α7). The fundamental composition of each dimeric interface is predominantly composed of the α3, α4, and α5 helices stemming from each monomer. Evidently, the asymmetric unit of the hetero-tetrameric complex harbors two-metal coordination sites, consisting of two Mg^2+^ sites and two Mn^2+^ sites.

In contrast, when considering the VapB15 antitoxin within the VapBC15 homodimeric complex, a significant portion of the protein remains invisible in the electron density map. This obscured segment includes approximately 40 residues in the N-terminus and around 10 residues in the C-terminus (Figure 5B). Insight into this phenomenon is gleaned from MALDI-TOF (matrix-assisted laser desorption/ionization time-of-flight) analysis, which elucidates that the protein underwent proteolysis during the crystallization process [54]. Consequently, the visualized region encompasses merely two short α-helices connected by a connecting loop.

The active site composition of VapC15 incorporates the presence of Mg^2+^ and Mn^2+^ ions. To validate its metal-dependent RNase activity, VapC15 toxin was synthesized by denaturing and refolding the VapB15 antitoxin within the VapBC15 complex. The structural integrity of the VapC15 toxin was affirmed through circular dichroism spectra analysis [54]. In an in vitro ribonuclease activity assay utilizing agarose gel electrophoresis, the VapC15 toxin displayed catalytic proficiency in degrading RNA derived from a specific *Escherichia coli* strain. However, when VapC15 was exposed to ethylene-diamine-tetra-acetic acid (EDTA), a metal-chelating agent, its catalytic activity on the same RNA substrate was nullified, providing conclusive evidence of its metal-dependent RNase activity [54].

### 2.5. VapBC26 System

**Figure 6:**
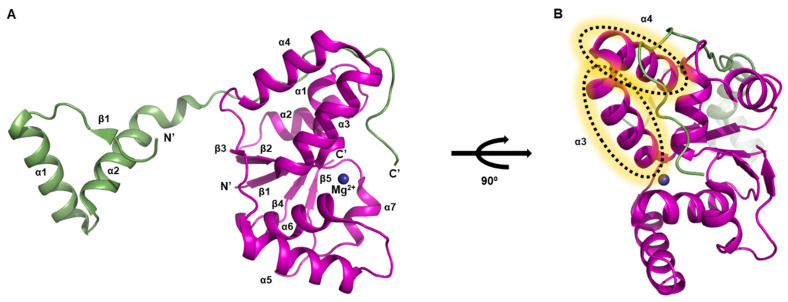
Heterodimeric assembly of *M. tuberculosis* VapBC26 system. Secondary structure nominations are displayed. Coordinate Mg^2+^ ion is also indicated. (**A**) Overall architecture of VapBC26 complex. (**B**) 90° rotated view of (**A**). α3 and α4 helices that used to design antimicrobial peptides are indicated.

The VapBC26 complex from *M. tuberculosis* contains four VapB26 antitoxins and four VapC26 toxins in a hetero-octameric assembly, as shown by the crystal structure (Figure 1E). The flexible hinge loop of the antitoxin acts as a hooked arm, wrapping around the toxin. In the structure of the VapBC26 homodimer, VapB26 binds to VapC26 along the deep valley formed by four α-helices (α1–α4) of VapC26 (Figure 6A). The VapC26 toxin adopts an α/β/α sandwich fold composed of seven α-helices and five β-strands, and the VapB26 antitoxin contains one β-strand and two α-helices with a topology of β1–α1–α2. As such, the detailed unit structure shows a similar pattern to the previously introduced VapBC systems in the previous subsection. However, in the study of the *M. tuberculosis* VapBC26 system, a peptide-based antimicrobial agent was generated using Mg^2+^-dependent RNase activity [49].

Based on the interaction between VapB26 and VapC26, several peptides were designed as potential inhibitors. These peptides were designed to mimic the binding interface of VapB26 and VapC26. Theoretically, these peptides could compete with VapB26 and VapC26 for binding, and therefore prevent the formation of the TA complex [49]. If the peptides bind with high affinity, the binding between VapB26 and VapC26 would be disrupted, and free VapC26 would become more predominant, leading to increased RNase activity. In fact, it has been confirmed that the ribonuclease activity of VapBC26 increases as a result of peptide addition [49,62]. Effective peptides were those that mimicked the binding region of VapC26, specifically the α3 and α4 helices (Figure 6B). It was found that VapBC26 exhibited increased activity upon the addition of the α4-mimicking peptide, compared to the addition of the α3-mimicking peptide [49]. Furthermore, modified peptides with a-helix stapling showed highly enhanced activity and cell permeability [62].

In the investigation of the VapBC26 system, efforts focused on designing peptidemimetics to target the VapC26 toxin and inhibit its interaction with the antitoxin VapB26 [49]. This led to the discovery of an inhibitory peptidomimetic, ‘V26-SP-8’, which specifically targeted the VapC26 α4 helix. ‘V26-SP-8’ was engineered from the initial peptide through hydrocarbon α-helix stapling, resulting in enhanced VapC26 activity even at significantly lower concentrations [62]. Circular dichroism spectroscopy confirmed the increased α-helical propensity of ‘V26-SP-8’, and isothermal titration calorimetry determined a dissociation constant (*K_d_*) of approximately 604 ± 18.2 nM for the VapB26-’V26-SP-8’ interaction. NMR spectroscopy revealed the binding mechanism between VapB26 and ‘V26-SP-8’. In experiments with *M. smegmatis*, fluorescence-labeled ‘V26-SP-8’ demonstrated uptake by bacterial cells and inhibited their growth effectively. Moreover, ‘V26-SP-8’ exhibited stability in human and mouse blood/serum/plasma, with a half-life exceeding three hours [62].

### 2.6. VapBC30 System

**Figure 7:**
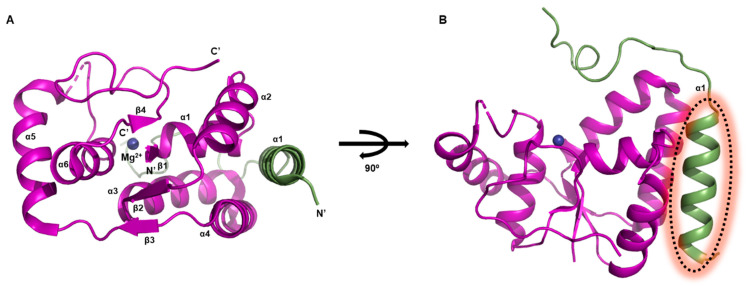
Heterodimeric assembly of *M. tuberculosis* VapBC30 system. Secondary structure nominations are displayed. Coordinate Mg^2+^ ion is also indicated. (**A**) Overall architecture of VapBC30 complex. (**B**) 90° rotated view of (**A**). α1 helix that used to design antibiotic peptide is indicated.

The *M. tuberculosis* VapBC30 complex exists as a hetero-tetramer in solution and consists of two tightly bound VapBC30 heterodimers (Figure 1F). Briefly, the VapC30 toxin is characterized by a PIN domain motif. It has an α/β/α sandwich fold consisting of four parallel β-strands in β4–β1–β2–β3 order with six α-helices (Figure 7A). VapB30 antitoxin offsets the enzymatic function of the cognate VapC30 toxin by forming the VapBC30 complex. Specifically, growth arrest or apoptosis effect caused by toxicity due to the expression of VapC30 was confirmed through bacterial cells [50]. The cells expressing VapC30 did not grow, but cells co-expressing VapB30 and VapC30 grew well under the same conditional as the control cells [50].

Using the structural features of the binding interface of VapBC30, it was possible to design a useful and effective antibiotic peptide. A certain peptide that includes the α1 helix of VapB30 antitoxin can mimic the binding of the VapBC30 complex (Figure 7B), resulting in the arrest of bacterial cell growth and eventually cell death [50,63]. The designed candidate peptides were optimized through the use of α-helix stapling technique [64]. The optimized peptides were able to successfully penetrate the bacterial cell membrane, and their minimum inhibitory concentration values were less than 6.25 μM [63].

The synthetically engineered peptide ‘V30-SP-8’ effectively penetrated *M. smegmatis* cells and surpassed the efficacy of the antibiotic vancomycin. These methods, guided by insights from *M. tuberculosis* TA systems, offer promise for the development of novel antibiotics tailored to combat antibiotic-resistant strains of *M. tuberculosis*. Given the lack of therapeutic agents targeting TA systems, the ‘toxin activation strategy’ holds potential for innovative antibiotic development, particularly against *M. tuberculosis* [50,63].

## 3. Concluding Remarks

Bacterial TA systems consist of two main components: toxins and antitoxins. Toxins target essential bacterial processes, while antitoxins neutralize their effects. These systems are categorized into various types based on the nature of the antitoxin and its interaction with the toxin. Type II TA systems, in particular, involve protein antitoxins that form complexes with protein toxins. Importantly, these type II systems lack counterparts in humans and are common in significant bacterial pathogens. This makes the protein products of type II TA systems promising candidates for the development of new antibacterial drugs. Extensive research has been conducted on the VapBC toxin–antitoxin systems in *M. tuberculosis*, and their crystal structures have provided valuable insights into their functions. The VapC toxins have a unique sandwich-like structure, while VapB antitoxins often possess DNA-binding motifs. The harmful effects of VapC toxins are due to their RNase activity, which is effectively countered by the strong binding of VapB antitoxins.

This review focuses on early efforts in structure-based drug development, specifically highlighting insights from the structural and biochemical aspects of type II TA systems. Antibiotic candidates, in the form of α-helix peptidomimetics, have gained attention due to their resistance against proteolytic degradation. Through stapling modifications of peptides, improved α-helical content and enhanced cell permeability have been achieved. The uptake of these peptides by cells was demonstrated, as they were completely removed from the cell surface during fluorescence-associated cell sorting (FACS) experiments after trypsin digestion. With the ongoing research into type II TA systems, including investigations into small molecule inhibitors, it is likely that new-generation antibiotics targeting these systems will emerge in the near future.

**Figure 8:**
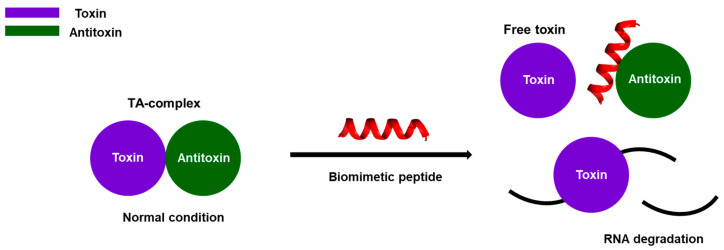
Schematic diagram of the therapeutic applications of biomimetic peptides in type II TA systems are described. The purple represents the toxin, which inhibits cell growth or causes cell death, while the green represents the antitoxin, which counters the toxin’s effects. In the absence of specific stimuli or stress, toxins remain stable and inactive due to the presence of antitoxins. However, a mimetic peptide can detach the antitoxin from the toxin, leading to cell death.

Biomimetic peptides, designed through rational design based on the structure of TA systems, have the potential to function as antibacterial agents [29]. In type II TA systems, it is possible to artificially activate the toxin by designing inhibitors that disrupt the interaction between the toxin and antitoxin (Figure 8). In this theory, the toxin within the TA complex remains non-toxic because the antitoxin obstructs the catalytic active site of the toxin. However, when an inhibitor interacts with its corresponding binding site, it can detach the antitoxin, leading to toxin activation. Furthermore, when the binding interface consists of α-helices, α-helix peptidomimetics are favored as antibiotic candidates due to their superior resistance to proteolytic degradation. Additionally in these cases, the application of stapling modifications to peptides can increase their α-helical content and enhance cell permeability, making them advantageous [49,62].

These designed peptides are based on unique TA complex proteins found only in bacteria. Therefore, they are predicted to have fewer side effects in humans when developed into drugs. Additionally, since the TA systems in specific bacteria vary structurally, it is expected that they can target and eliminate only pathogenic bacteria, not harming beneficial ones in humans. However, there is a concern that a moderate degree of toxin activation might lead to the formation of persister or dormant cells, contributing to chronic infections. Therefore, it is essential to develop strategies to control the dosage to prevent cells from entering a dormant stage or render them susceptible to antibiotic drugs. As these challenges are gradually overcome, we can anticipate the development of new-generation antibiotics in the near future by targeting the TA system.
